# LIFE EXPECTANCY WITH CHRONIC DISEASE AMONG OLDER MEXICAN AMERICANS

**DOI:** 10.1093/geroni/igz038.931

**Published:** 2019-11-08

**Authors:** Kerstin G Emerson, Anqi Pan, Hanwen Huang

**Affiliations:** University of Georgia, Athens, Georgia, United States

## Abstract

The Hispanic Paradox research consistently shows a survival advantage among Hispanics, where they have significantly longer life expectancy compared to non-Hispanic Whites. However, less is known about the quality of these additional years of life. Our goal was to calculate life expectancy for older Hispanics in the US, and to determine what proportion of their lives they could expect to live with chronic conditions. We used data from 2004-5 of the Hispanic Established Population for the Epidemiological Study of the Elderly (Hispanic-EPESE), linked to vital status data through 2016. To determine life expectancy with/without chronic conditions, we used Sullivan’s method. Chronic conditions included: diabetes, stroke, heart attack, arthritis, hypertension, and cancer. Finally, we mapped life expectancy without chronic conditions across neighborhood characteristics. The sample consisted of 2,069 Mexican Americans aged 75 and older. Results showed that at age 75, Mexican Americans could expect to live a large portion of their lives with at least one chronic condition (88%). The largest proportion of life lived with disease was for hypertension (61%), arthritis (59%), and diabetes (33%). There was no pattern by neighborhood characteristics for disease-free life expectancy. Gender differences could not be examined because of small sample sizes. Our findings show that regardless of neighborhood characteristics, Mexican Americans can expect to live a high proportion of their life with at least one chronic condition. This is particularly high for hypertension, diabetes, and arthritis. It is important to consider quality as well as quantity of life when exploring the Hispanic Paradox.

